# Safety of simultaneous bilateral intravitreal versus unilateral anti-vasculo-endothelial growth factors injection in an operating room setting

**DOI:** 10.12669/pjms.38.8.5125

**Published:** 2022

**Authors:** Irfan Kabiruddin Jeeva, Sidra Masud, M.A. Rehman Siddiqui, Hadees Murad Fahad

**Affiliations:** 1 Dr. Irfan Kabiruddin Jeeva, FRCOphth (UK), FEBO (EU), CCT (UK), Department of Ophthalmology and Visual Sciences, Aga Khan University Hospital Karachi, Karachi, Pakistan; 2 Dr. Sidra Masud, MBBS, Department of Ophthalmology and Visual Sciences, Aga Khan University Hospital Karachi, Karachi, Pakistan; 3 Dr. M. A. Rehman Siddiqui, FRCOphth (UK), CCT (UK) Consultant Ophthalmologist, Department of Ophthalmology and Visual Sciences, Aga Khan University Hospital Karachi, Karachi, Pakistan; 4 Dr. Hadees Murad Fahad MBBS. Layton Rahmatullah Benevolent Trust (LRBT) Teaching Eye Hospital, Karachi, Pakistan

**Keywords:** Endophthalmitis rate, anti-VEGF injections, anti-VEGF injection safety, Bilateral vs Unilateral anti VEGF

## Abstract

**Objectives:**

Chorioretinal diseases requiring the use of anti-vascular endothelial growth (anti-VEGF) injections often occur in both eyes simultaneously. This can necessitate injecting both eyes together rather than one eye at a time. The purpose of the study was to determine whether simultaneous bilateral intravitreal injections of anti-VEGF agents are safe when administered in an operation theatre setting.

**Methods:**

Retrospective review of data was conducted. Single center study conducted in a tertiary care hospital in Karachi Pakistan. Approximately 30,000 eyes that received anti-VEGF injection during a 10-year study period were included (March 2008-February 2018). Patients who were lost to follow up prior to completion of treatment were excluded. Consecutive sampling technique was employed. The patients who received bilateral anti-VEGF injections were analysed separately from the ones who received unilateral injections. All injections were administered in operating theatre setting. The rate of endophthalmitis was measured in each group.

**Results:**

A total of 30,258 injections were administered of which 15,338 were bilateral injections. Four cases (4/30,258, 0.013%) of endophthalmitis occurred during the study period. Only one case (1/15,338, 0.0065%) of endophthalmitis occurred after the administration of simultaneous bilateral anti-VEGF injections.

**Conclusions:**

Administration of simultaneous bilateral anti-VEGF injections was safe in our population.

## INTRODUCTION

Chorioretinal diseases refer to disorders of the retina and vascular choroid layer of the eye. The most prevalent of these include ischemic retinopathies such as diabetic retinopathy and retinal vein occlusion in which retinal vein damage results in ischemia of the retinal vasculature.^1^ The other prevalent type is age-related macular degeneration (AMD), which involves damage to the outer retina.^1^ All these conditions are marked by the stabilization of Hypoxia inducible factor-1 (HIF-1) leading to the upregulation of several gene products, primarily, Vascular Endothelial Growth Factor (VEGF) resulting in Neovascularization (NV).^1^ Newly formed blood vessels are often oedematous thereby inflicting damage to the retina and resulting in symptoms such as distorted vision, floaters or even blindness.^1^

With regard to the pathophysiology described above, anti-vascular endothelial growth factor (anti-VEGF) agents have been identified as a revolutionary treatment plan for the management of chorioretinal diseases.^2^ They act by inhibiting the action of VEGF thus controlling the effects caused by NV. Commonly administered anti VEGF agents include aflibercept (Eylea; Regeneron, Tarrytown, New York.), ranibizumab (Lucentis; Genentech, San Francisco, California.), and bevacizumab (Avastin; Gennetech).

Drug delivery to the posterior eye segment can be challenging and is mostly achieved via intravitreal injections.^3^ Chorioretinal diseases often affect both eyes; hence, bilateral intravitreal injections of anti-VEGF are required. Injecting both eyes simultaneously, in one sitting, potentially decreases the burden on both the patient and the physician, as well as that of the caregiver. According to a survey, nearly half (46%) of retina specialists in the United States administer bilateral injections.^3^

Intravitreal injections are associated with complications including the risk of development of endophthalmitis.^4^ Endophthalmitis is a severe inflammatory process affecting the vitreous cavity, retina and uveal apparatus of the eye that may progress to complete blindness. Other complications may include elevated Intra-Occular Pressure (IOP) or transfer of silicone vesicles or protein aggregates from syringes to eye disturbing its visual acuity.^5^ Therefore, one can argue regarding the safety level of bilateral and unilateral intravitreal injections.

At our tertiary care centre in Karachi, Pakistan, anti-VEGF agents aflibercept, ranibizumab and bevacizumab are currently used for the management of chorioretinal conditions. Anti-VEGF agents are administered both unilaterally and bilaterally. The aim of this study was to determine whether the rate of endophthalmitis occurring after intravitreal anti-vascular endothelial growth factors (VEGF) is different for simultaneous bilateral injections and unilateral injections when all factors are kept constant.

## METHODS

This retrospective study was carried out at Aga Khan University Hospital Karachi, Pakistan. Approval was taken from the institute’s Ethical Review Committee (ERC) prior to the study for retrospective review of data. As it was a retrospective review the ERC waived full review (ERC #: 2020-3436-11633, Date: 24^th^ August 2020). The study employed a cross sectional design. Patients were identified using the hospital electronic coding system by using convenience sampling. Data for all patients was obtained from the medical records. Those patients who had received aflibercept, bevacizumab and/or ranibizumab in the last 10 years (1^st^ March 2008 – 28^th^ February 2018) were included in the study. Patients receiving other intravitreal injections such as corticosteroids were excluded from the study.

All intravitreal injections were administered following the hospital protocol in the operating room (OR) under aseptic conditions. All members of the OR used a mask. Each patient was first prepped by a trained OR technician after which Proxymetacaine hydrochloride 5% drops were instilled in the conjunctiva. The eye, including the eyelid skin and eye lashes, was cleaned with povidone –iodine 5% solution two minutes prior to the procedure, followed by sterile draping. A sterile speculum was inserted to direct the lashes away from the eye. Proxymetacaine hydrochloride 5% drops were applied again after speculum removal. Using a calliper, the distance from the limbus was measured; 3.5mm for pseudophakic and 4mm for phakic patients.

An ophthalmologist wearing sterile gloves then administered the injection. For simultaneous bilateral injection the same preparation was done. The anti-VEGF for each eye were prepared in the pharmacy under sterile conditions using different batch numbers for each injection. A new set of equipment; including gloves and sterile speculum was used for each eye of each patient. Following all injections one drop of topical antibiotic (moxifloxacin) was instilled onto the conjunctiva, and all patients were instructed to use post injection topical moxifloxacin eye drops four times a day for 5-7 days. Patients were called for follow up assessment within one week. However, as per each surgeon’s discretion they were minor alterations between the surgeons in the post-operative care such as the antibiotic given prophylactically. Cleaning with povidone Iodine 5% and administration of the injection in OR setting was constant for all surgeons throughout the study period. Statistical analysis was carried out using SPSS version 20.

## RESULTS

**Fig.1:**
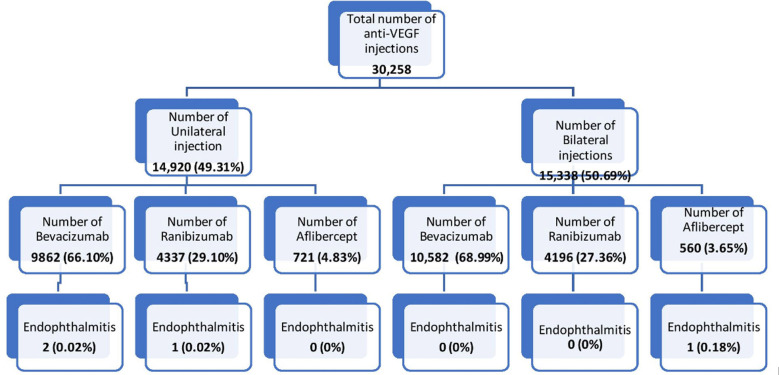
Flowchart depicting the distribution of injections and subsequent endophthalmitis rates.

Data was obtained over a 10-year period. The patient selection and outcomes are shown in Fig.1. A total of 30,258 intravitreal anti-VEGF injections were administered over this period, out of which 14,920 (49.31%) were unilateral injections and 15,338 (50.69%) were bilateral injections. In the unilateral group, 9,862(66.1%) patients received bevacizumab, 4,337 (29.1%) received ranibizumab and 721 (4.83%) received aflibercept.

In the bilateral group, 10,582(68.99%) patients received bevacizumab, 4,196 (27.36%) received ranibizumab and 560 (3.65%) received aflibercept. (Fig.1) They were a total of 7,170 eyes which were treated at our institution in this 10-year period. The mean number of injections per eye was 4.22 ± 4.838 (95% CI).

Four cases of endophthalmitis were identified. (Table-I) Hence, the risk of endophthalmitis at our tertiary care center was calculated to be 0.013 % (4 of 30,258) for all injections. All four patients who developed endophthalmitis were male, between the ages of 36 and 78. One of the patients was a known diabetic and was being treated for proliferative diabetic retinopathy. The others were receiving treatment for cystoid macular oedema, vitreous hemorrhage and age-related macular degeneration respectively. Out of the four patients who developed this complication, two had received bevacizumab injections, one had received ranibizumab and one had received aflibercept.

**Table-I:** Summary of patients with clinically suspected endophthalmitis

Case	Age	Baseline Diagnosis	DM	Medication	Preinjection Visual Acuity	Visual Acuity at The Time Of Endophthalmitis	Intraocular Culture Results	Final Visual Acuity
1	36	CME	No	Ranibizumab	20/25	20/150	Culture not sent	20/25
2	55	PDR	Yes	Aflibercept	20/50	HM	Strep. Group D	20/150
3	54	Vitreous Hemorrhage	No	Bevacizumab	N/A	HM	Culture not sent	HM
4*	78	ARMD	No	Bevacizumab	N/A	N/A	Culture Negative	N/A

DM: Diabetes Mellitus, CME: Cystoid macular edema, HM: Hand Movement, PDR: proliferative Diabetic retinopathy, ARMD: Age related Macular Degeneration.

N/A, Not available. *Patient records incomplete.

For simultaneous bilateral injections there was only one case (0.0065%) of unilateral endophthalmitis, whereas for unilateral injections they were 3 cases (0.02%). After the management of endophthalmitis, visual acuity of two patients improved to 20/25 and 20/150 respectively. The visual acuity of one patient, however, remained to only appreciating hand movements.

## DISCUSSION

Infectious endophthalmitis is one of the most serious complications of intravitreal anti-VEGF administration. Pathogens gain access to the vitreous cavity during or after the administration of intravitreal anti VEGF agents causing this unfortunate reaction. Generally, the surrounding environment and the ocular surface are the main sources of contamination. However, at times contaminated instruments, needles or the drug itself can also become a potential source. Adapting to better safety standards by adhering to proper antiseptic injecting techniques is a step towards decreasing the incidence of endophthalmitis and its serious repercussions.^6^

Literature estimates the incidence of post injection endophthalmitis of between 0.007% and 0.16% per injection.^7^-^12^ The risk of endophthalmitis occurring in simultaneous bilateral injections is between 0.00% and 0.48%, according to literature.^7^,^13^-^16^ To minimize the risk of this severe complication, several protocols have been proposed.

Previously conducted local studies have revealed a relatively higher rate of endophthalmitis after intravitreal injections. Khaqan et al reported a post bevacizumab injection endophthalmitis to be 0.134%.^17^ Two other studies studying the complications after anti VEGF revealed similar rates of 0.11% and 0.10% respectively.^18^,^19^ The rate of endophthalmitis per intravitreal injection of anti VEGF agents at in our hospital was four of 30,258 (0.013%), in an OR setting, in the presence of positive pressure ventilation. Globally the rate of incidence of endophthalmitis is reported to be between 0.028% to 0.04% per injection.^6^,^20^ This rate is also comparable to other studies which have reported a similar rate in the administration of intravitreal anti-VEGF agents in an OR setting.^3^,^7^-^12^,^21^,^22^ A comparison of the rate of incidence of endophthalmitis in other studies vs our institution is shown in Table-II.

**Table-II:** Incidence of endophthalmitis after intravitreal injection.

Study	Year of publication	Country	Medication	Number of cases of endophthalmitis	Number of injections	Rate of infection (per injection), n(%)	Setting
Moshfeghi^4^ et al	2011	USA	Bevacizumab and ranibizumab.	12	60,322	0.02%	Office
Khaqan^17^ et al	2012	Pakistan	Bevacizumab	7	5189	0.134%	Operating theatre
Abell^12^ et al	2012	Australia	Bevacizumab and ranibizumab.	4	3,376	0.12%	Office
Abell^12^ et al	2012	Australia	Bevacizumab and ranibizumab.	0	8,873	0.00%	Operation theatre
Rayess^11^ et al	2016	USA	Bevacizumab, ranibizumab and aflibercept.	183	503,890	0.036%	Office
Ruão^7^ at al	2017	Spain	Ranibizumab and aflibercept	1	8,172	0.012%	Sterile room
Freiberg^8^ et al	2017	Denamark and Switzerland	Bevacizumab, ranibizumab and aflibercept.	10	134,701	0.007%	Operation theatre
Farooghian^9^et al	2017	Canada	Bevacizumab, ranibizumab and aflibercept.	26*	63,183	0.041%	Office
Daien^10^ et al	2017	Australia, New Zealand and Switzerland	Bevacizumab, ranibizumab and aflibercept.	18	88,150	0.02%	Not available
Khaqan^19^ et al	2019	Pakistan	Bevacizumab, ranibizumab and aflibercept.	3	2854	0.105%	Operating theatre
Current study	2018	Pakistan	Bevacizumab, ranibizumab and aflibercept.	4	30,258	0.013%	Operation theatre

*4 more cases of endophthalmitis were suspected in this study but they were excluded from the final result as they occurred during the first 24hrs of the injection and were considered sterile endophthalmitis.

At our institution anti-VEGF agents are also administered bilaterally in one sitting. When compared to the incidence of endophthalmitis occurring in unilateral injections (0.02%), bilateral has a lower rate (0.0065%) when all other factors are kept constant. Recent work by Borkar et al. reports safety of bilateral same-day intravitreal anti-VEGF treatment, supporting the observations of our study.^23^ A recent study conducted in Spain reported no incidence of endophthalmitis occurring in 1,612 injections administered bilaterally.^7^ Other studies have also reported a similar rate in simultaneous bilateral injections (Table-III). The ratio of the studies conducted in the operating room setting vs the office room was comparative (1.2:1). The endophthalmitis rates were not greater is any one setting and ranged between 0% to 0.105%. (Table-III)

**Table-III:** Rate of endophthalmitis after simultaneous bilateral intravitreal injection.

Study	Year of publication	Country	Medication	Number of cases of endophthalmitis	Number of injections	Rate of infection (per injection), n (%)	Setting
Bakri^15^ et al	2009	USA	Bevacizumab and ranibizumab.	1	208	0.48%	Office
Lima^16^ et al	2009	USA	Bevacizumab and ranibizumab.	2	3,068	0.065%	Office
Davis^14^ et al	2010	USA	Bevacizumab and ranibizumab.	0	1,322	0.00%	Not available
Mahajan^13^ et al	2011	USA	Bevacizumab and ranibizumab.	0	904	0.00%	Office
Ruão^7^ et al	2017	Spain	Ranibizumab and aflibercept	0	1,612	0.0%	Sterile room
Grzybowski^25^et al	2018	USA	Bevacizumab, ranibizumab and aflibercept.	28	101,932	0.027%	Office
Current study	2018	Pakistan	Bevacizumab, ranibizumab and aflibercept.	1	15,338	0.0065%	Operation theatre

The incidence of endophthalmitis has perhaps remained low due to the safety protocol that is in place while administering the intravitreal injection. Perhaps limiting the administration of the injection in a positive pressure ventilation OR setting has further reduced the incidence of endophthalmitis at our institution. A retrospective study conducted in India reported an endophthalmitis rate of 0.050% for anti-VEGF injections administered in ambulatory surgical units which included both in-office setting as well as an OR one.^21^ Meanwhile, Tabandeh et al, a retrospective study, did not report a significant difference in the incidence of endophthalmitis between the two settings.^22^

### Limitations:

The study was retrospective in nature. Hence, some records had been lost over the years resulting in our clinical information to be incomplete. Our data, however, was rechecked using the billing records, thus it is unlikely that any case of endophthalmitis is missing. Another limitation of our study was the inability to assess possible systemic complications of intravitreal injections such as high blood pressure, extraocular hemorrhage, stroke, and myocardial infarction, as a substantial proportion of our patients receive treatment outside our hospital, thus limiting our access to their information.

### Strength of the study:

The study included data from the past 10 years consisting of 30,258 anti-VEGF injections, which is the largest data analyzed from within Pakistan. Hence, it is perhaps a clear depiction of the rate of endophthalmitis occurring in our country as well as the developing world. Furthermore, the procedure carried out for bilateral intravitreal injections was as per the recommendations of The Euretina Expert Consensus,^24^ which discourage the reuse of equipment for each eye. Even though conducted in a single centre, the study takes into account the real-life experience of patients receiving anti-VEGF treatment rather than being a protocol driven study. Multiple surgeons administering the injections limit any bias and strengthen the results our study shows.

## CONCLUSION

The present study shows that the administration of simultaneous bilateral anti VEGF injections is safe in our setting.
